# The insensitivity of ‘sensitive care’: the bureaucracy of pregnancy tissue disposal in England, UK

**DOI:** 10.1080/13648470.2024.2416804

**Published:** 2024-10-24

**Authors:** Susie Kilshaw

**Affiliations:** UCL Anthropology, London, UK

**Keywords:** Miscarriage, pregnancy ends, normativity, personhood

## Abstract

The practices surrounding pregnancy ends and pregnancy remains shift and change depending on the cultural and historical context. Based on ethnographic research in one group NHS Hospital organisation in England, the paper explores what practices around pregnancy remains reveal about the values afforded the material in different contexts by different actors and the moments when these intersect. It argues that framing miscarriage as bereavement helps to structure caregiving in clinical settings and that clinical practices produce foetal personhood in ways that may not be in keeping with women’s notions of their pregnancy material. It illustrates that hospital practices contain notions of value which become legitimated as the appropriate approach with consequences for normativity. This may lead to women feeling isolated and abnormal when their approach is at odds with that of the clinic. Through an exploration of how women encounter and negotiate disposal practices, the paper argues that current practice requires revision to flexibly respond to diversity but also shifting meaning and values attributed to these experiences and materials.

## Introduction

The end of a pregnancy engages a woman’s relationship with her body, her self, her pregnancy material and/or foetus. The practices surrounding miscarriage, including how pregnancy material is handled, shift and change depending on the cultural and historical context, but also location. Based on ethnographic research in a National Health Service (NHS) Trust^1^ in England, I explore what practices reveal about the values afforded the material in different contexts by different actors and the moments when these intersect. Interrogating such practices allow for an exploration of the boundaries of life and death and categories of personhood. There has been a cultural and clinical shift in how miscarriage is approached and managed with a move towards greater sensitivity, however, the paper argues that elements of policy and care are not experienced as sensitive by most women. The paper builds on interdisciplinary scholarship that reveals that aligning pregnancy loss as bereavement structures caregiving in clinical settings (Fuller and Kuberska 2022) and that clinical practices produce foetal personhood (Middlemiss 2021, 2024, Kuberska 2020) that may not be in keeping with women’s notions of their pregnancy and pregnancy material. It further argues that hospital practices contain notions of value towards pregnancy materials which become legitimated as the appropriate approach with consequences for normativity (Kuberska 2020).

Sociocultural studies of reproduction widely use the language of reproductive and pregnancy loss; my research is situated amongst a growing body of work which suggest ‘pregnancy ends’ better captures the complexity and nuance of women’s experiences. Incorporating abortion as well as spontaneous pregnancy endings, the term removes intentionality as the defining element (in the UK context, see Middlemiss 2024, Austin et al. 2021; Austin and McGuinness 2019; Kuberska et al. 2020) and assumptions around reactions to types of ends. The term is more inclusive of diversity of pregnancy ends including molar and ectopic pregnancies as well as the multiplicity of reactions to such experiences, as revealed in the interviews. Such inclusivity is important, as women may move between categories, as experienced by a participant of my previous research project who was devasted when she miscarried a day before her appointment to terminate the pregnancy and many women encounter different types of pregnancy endings. A key finding of my research is that women’s experiences and responses to pregnancy ends are diverse and culturally contingent (Kilshaw 2020a, 2020b, 2020c; Kilshaw and Borg 2020; Middlemiss and Kilshaw 2023; Kilshaw 2024a). Throughout this paper I use the terms that my interlocutors used or that were outlined in clinical documents (‘miscarriage’, ‘pregnancy tissue’, ‘baby’, ‘pregnancy remains’), but sometimes refer to the more general ‘pregnancy material’ when their language was ambiguous. This is to avoid the connotations that ‘pregnancy remains’ may provoke with its proximity to ‘human remains’. I highlight that women did not necessarily see the pregnancy material as ‘remains’ and to reflect that it may or may not contain a foetus or foetal tissue.

The paper considers the construction of the foetus in different contexts and temporalities, as something processual and defined by its interactions. There is no ‘natural’ or ahistorical foetus: all foetuses are socially, culturally, and politically constructed and construction varies depending on who is attributing meaning (Casper 1998). Pregnancy materials may be viewed as waste, an anatomical specimen, or a human corpse with approaches to and regulation of their handling being historically, culturally, and geographically contingent (Morgan 1999, 2002). By exploring meaning attributed to the foetus and/or pregnancy material by clinical disposal practices and women’s encounters with these, the paper contributes to scholarly work exploring the ontologies of the not alive foetal body (Middlemiss 2021, 2024; Fuller and Kuberska 2022; Smidova 2019), the changing significance of this material in a variety of ethnographic settings (Charrier and Clavandier 2019; Memmi 2011) and ontologies of pregnancy materials in different ethnographic settings (Kilshaw 2017; Lupton 2013; Kaufman and Morgan 2005).

I explore how women encounter and negotiate disposal practices, first describing how women’s experiences of miscarriage are informed by location. I argue that hospital practices compel women to encounter their pregnancy tissue and to make moral decisions about its ontological status in a way that is not always present in domestic settings. Elsewhere I have described how most women find practices around pregnancy tissue disposal to be unexpected, inappropriate and at times distressing (Kilshaw 2024a); I expand this discussion and argue that divergence in attributions of personhood is at the heart of negative experiences. In some cases, ‘sensitive care’ may be experienced as insensitive. Clinical practices and the meaning they contain become legitimated as the appropriate, right, moral approach with consequences for normativity (Kuberska 2020) leading women to feel abnormal when their approach is at odds with that of the clinic. The NHS has made consistent and concerted efforts to implement evidence-base care in general and, thus, hospital practices are likely assumed to be based on patient values, clinical expertise, and best evidence. Approaches to pregnancy material have shifted and will continue to transform: I argue that current practice requires revision to flexibly respond to diversity and shifting meaning of experiences and materials. The paper seeks to contribute to medical anthropological theories about the politics of life and death and what and, importantly, who defines what is a person and a mother. This is particularly salient when one considers how claims about the ontological status of foetuses and pregnancy materials have significant implications for a variety of practices such as abortion; embryo creation, storage and disposal; their use for research purposes; fertility treatments amongst others.

## Methods

The paper is derived from data from ethnographic research which included extensive fieldwork including participant observation and semi structured interviews with women experiencing miscarriage (n37) and those involved in their care (n38) over a 20-month period between February 2021 and September 2023.^2^ Women were recruited at one NHS Trust in England. Participation was offered on posters and flyers displayed in the site and by clinic staff familiar with the study who handed the study information sheet to women. Twenty-seven women self-referred and after expressing interest *via* email or phone call, potential participants were again sent the information sheet and consent form and given an opportunity to ask questions. Informed consent to participate was sought and recorded prior to interview. Women were interviewed during or soon after their miscarriage and follow up interviews were conducted 3–6 months later. Whilst the research included miscarriages up to 23 weeks and 6 days, all but one participant experienced first trimester miscarriage, and this is the focus of the paper. Semi structured interviews explored interactions with pregnancy materials, the meaning it holds, and experiences of disposal in both clinical and domestic settings. The research design was informed by previous research into experiences of miscarriage in England (Kilshaw 2020a, 2020b).

Most interviews were conducted in person, but due to COVID-19 restrictions a small number were conducted online. Interviews lasted 45–90 minutes. Individual, semi-­structured interviews were conducted as ‘guided conversations’ (Lofland and Lofland 1984) and respondents were encouraged to give their own accounts and meanings in relation to the main research questions; their experience of the remains of miscarriage broadly defined, and their experience associated with disposal of pregnancy tissue. Fieldnotes were recorded after each interview. Audio recordings were professionally transcribed verbatim. Transcripts were reviewed for accuracy and familiarity. Fieldnotes and interview transcripts were analysed using thematic analysis approach, the method was iterative and based on a grounded theory approach (Strauss and Corbin 1990).

Observations were not recorded, and no personal information was collected. Staff were informed of the research and invited to participate. Participating staff would provide women with the study information and seek verbal consent for observation. If both agreed the researcher would be invited into the session. Some participants took part in both observations and interviews. All elements of miscarriage care were observed including: assessment, diagnosis, ultrasound scanning, medical and surgical management of miscarriage, consenting process for miscarriage management and remains disposal, and follow-up appointments. Disposal pathways, memorialisation activities, hospital ceremonial disposal were all observed. Interviews with 38 health professionals involved in the care of miscarriage or handling of pregnancy remains were conducted.

Between April 2020 and September 2022, 27 women made contact agreeing to participate and were subsequently interviewed. No participants withdrew consent. 10 women who participated in my previous research and who were treated in the same NHS Trust were interviewed to explore long term remains and residues of miscarriage including ongoing acts of memory and memorialisation and/or their absence and to probe their experience of pregnancy remains disposal when they miscarried six years prior. Participants volunteered a variety of reproductive histories, and many had experienced earlier pregnancy endings including miscarriages, ectopic pregnancies, abortions, and one stillbirth. These experiences were often used by participants as points of resonance or comparisons and several participants had had experiences of both home miscarriage and clinically managed miscarriage.

The research was granted full ethics approval by the UK Health Research Authority.

## Location, agency, and choice

### Miscarriage and its remains at home

If pregnancy tissue emerges in a domestic setting prior to 23 weeks and 6 days gestation, a woman can choose what happens to that material. Fifty percent of the women I interviewed in England for my current and past research miscarried at home. Most women (65%) flushed the material down the toilet or put it in the bin. Some women collected their pregnancy material or retrieved it from the toilet, most commonly because they thought the health professionals might inspect it for diagnostic reasons. Some women thought that the material might need to be examined to assess whether the miscarriage was complete whilst others hoped the tissue might be tested to diagnose cause, particularly in cases of recurrent miscarriage. Others collected the material because they didn’t want to flush it away and instead wished to dispose of it through other means. Of the 67^3^ women I have interviewed in England who miscarried at home, 16% collected all or some of the pregnancy tissue for the purpose of ceremonial disposal either by home burial (N4) or private cremation (N1), thus a minority of women treated their pregnancy remains in this way.

What women do with their pregnancy tissue in their home can remain private. During interviews many women expressed confidence in this means of disposal whilst others spoke of uncertainty, reservations, or regret. Some women questioned their decision to treat the material as waste. When probed, most suggested this was in response to perceived scrutiny of others, including me, the researcher. At times women questioned their approach when encountering public discourses around pregnancy ends. In other cases, women who miscarry at home question their disposal method and its meaning when experiencing clinical practices. A small number of women had expelled and flushed the pregnancy material at home, but required clinical interventions to remove remaining tissue; expressing surprise that the tissue removed in the clinic required a discussion and decision about disposal. Women encountering hospital disposal practices for a subsequent miscarriage following a previous miscarriage at home similarly expressed surprise and/or disquiet, as will be discussed in the next section. As Melanie, highlighted,

[If I had miscarried at home] it would have gone in the bin like my sanitary towel. Like all the other ones, I just treated it like a period sort of thing.

The site of the expulsion of the physical material of miscarriage has a significant role in how it is interacted with in surprising ways. Women’s experience of miscarriage and the disposal of pregnancy material at home provide interesting insight into privacy, autonomy, and performance. For many women flushing pregnancy material is unproblematic; for others it is straightforward until other approaches are encountered, which is the subject of forthcoming publications. In the current reproductive milieu, men and women, parents, and parents-to-be, are faced competing, sometimes contradictory, discourses regarding not only how they should plan and execute their reproductive activities, but also how, to whom, and in what ways they need to ‘account’ for these actions (Faircloth 2013). The location of a miscarriage impacts privacy and agency: women miscarrying in domestic settings are not presented with bureaucratic hierarchy and disposal options faced by those whose pregnancy tissue emerges in clinical settings. These women are forced to encounter their pregnancy in particular ways and are confronted with choices, requirements to act, and governance of this material, as I will discuss in the next section.

### Miscarriage and its remains in the clinic

While some women experience the spontaneous expulsion of the pregnancy many women seek or require medical intervention. In the UK, women are offered a choice of three options, guided by their preference or situation^4^: expectant management (waiting for the miscarriage progress on its own, which occurs in around 60% of cases), medical management (prescribed medication dilates the cervix and causes the uterus to expel pregnancy tissue with a success rate of around 80%), and surgical management (removal of the tissue during a minor operation involving inserting a small suction tube into the womb to gently remove any tissue). Regardless of the chosen management option, it is possible some tissue will remain, and further treatment may be needed.

The majority of pregnancy tissue that appears in clinical settings does so as the result of surgical management of a pregnancy end. Local clinical approaches are informed by national guidance, particularly that issued by the Human Tissue Authority (HTA) in 2015 and regulated by UK law. While a clear set of legal rules surrounds the disposal of the body of a baby born dead after 24 weeks, the law governing the disposal of remains prior to this gestational age is less clear (Austin and McGuinness 2019, 139). The HTA ‘Guidance on the disposal of pregnancy remains following pregnancy loss or termination’ outlines that women should be given options (burial, cremation, and sensitive incineration) for the disposal of their pregnancy material (HTA 2015), although how this has been interpreted and executed varies between local NHS settings (for full discussion of development of these practices see Kilshaw 2024b; McGuinness and Kuberska 2017). In the Trust where the research was based, the practices were revised following the issuance of guidance with updated practices in place in 2018. Women are given three options: hospital disposal in the form of communal burial or releasing the material for private arrangement either to a funeral director or directly to the woman. A form ‘Consent for respectful disposal of tissue following a miscarriage or surgical ending of pregnancy’ framed the discussion and required a signature of the woman to document their choice.

Historically, pregnancy tissue was routinely treated as clinical waste and incinerated with women not involved in decisions about disposal. However, a cultural shift in the way pregnancy loss was approached and following a series of scandals in the 1990s and 2000s this practice was no longer deemed appropriate. The Alder Hey organs scandal involved the unauthorised removal, retention, and disposal of human tissue between 1986–1996; the official report, the Redfern Report (2001), revealed that foetuses and stillborn babies were stored in 210 NHS facilities and that Liverpool’s Alder Hey Children’s Hospital stored 1,500 miscarried, stillborn, or aborted foetuses without consent. In response to the report’s findings, Parliament established the HTA in 2005. In 2014 the Dispatches programme (24/03/14) highlighted the routine incineration of pregnancy remains without patient consent at some hospitals. In response, the HTA were sought to develop guidance on the disposal of pregnancy tissue, despite falling outside the scope of its regulatory remit due to such material being legally considered the woman’s tissue.

The ‘Death before Birth’ (DBB) project explored NHS practices around pregnancy remains disposal including the impact of the HTA guidance and found over half (44 of 81) of NHS trusts^5^ evaluated had changed their policy following issuance of the guidance (McGuinness and Kuberska 2017) whilst most trusts had framed pregnancy remains as something to be handled sensitively prior to 2015. Although consent for disposal is not required, due to pregnancy remains being legally considered the woman’s tissue, most NHS trusts use such forms to ensure an audit trail. Most women experiencing miscarriage encounter this practice in relation to surgical management of their miscarriage. The timing of the ‘consenting’ for remains disposal typically occurs at the same time as the consent for the surgical procedure, although it might happen prior to or after. Any pregnancy material that emerges or appears in a clinical setting will prompt the same process.

During my fieldwork, it soon became clear that discussions around pregnancy remains disposal and the consenting process caused discomfort and unease with both health professionals and patients. Most participants found the process and form itself as ‘jarring’ and not in keeping with the sensitive, caring, and responsive care they received (Kilshaw 2024a). It is important to note that women were not suggesting that health professionals were insensitive, but rather it was the bureaucracy itself that was not in keeping with otherwise sensitive care. The practice constrained interactions between women and health care providers and compelled them to engage in a way that was contrary to a relationship of care. During interviews with health care professionals, this divergence also emerged, as many described finding the process and form as problematic. Disposal practices emerged as element of care that commonly caused upset, tension, and friction when women did not want to discuss or be confronted with the pregnancy material and engaging with its meaning.

## Ambiguities of foetal personhood

What seems to be at the heart of discomfort is the tensions around messy foetal personhood in the face of pregnancy ends as reproduced by women, their partners and family, as well as clinical and cultural contexts. Personhood is both present and acknowledged as well as missing or denied. As evidenced by the convention that pregnant women are known as ‘mums’ at the same time as they may refer to themselves as ‘going to be a mum’ who then may be considered neither when a pregnancy ends. This ambiguity is part of lived experience as one moves through the experience of pregnancy and pregnancy ends. However inflexible bureaucratic procedures are unable to accommodate such nuance and ambiguity.

Aimee Middlemiss (2021) has revealed how the governance of the dead foetal body once outside the person’s body in England is not only incoherent, but produced inconsistencies in personhood which are navigated by those experiencing second trimester pregnancy ends. Scholars have revealed the contested nature of what is lost: a possible future, pregnancy tissue, a baby, a person (Middlemiss 2021) and how foetal personhood can be negotiated through various practices, such as the use of material goods by women in the US (Layne 2000, 2003). My research engages with previous work which shows how disposal practices and access to funeral rites can lend personhood to the pre-term foetus, as shown in France (Charrier and Clavandier 2019) and the UK (Kuberska 2020), adding further weight to the work of anthropologists who reveal how biomedical discourses and practices produce personhood (Mitchell 2016). In the UK (and other contexts) there has been the emergence of practices that favour foetal personhood in the approach to miscarried tissue.

Elsewhere I have written about how women were unprepared for disposal practices and had not considered their need (Kilshaw 2024a). Dominant themes are that it was inappropriate in relation to pregnancy duration, stage of foetal development and/or miscarriage trajectory (Kilshaw 2024a), which I develop in the next section. I explore how varying meaning of pregnancy tissue and foetal personhood results in discomfort and, at times, distress. Pregnancy ends involve the boundaries of personhood; anthropological theories about when it begins and ends are relevant (Kaufman and Morgan 2005, Layne 2022). The first anthropological work focusing on pregnancy loss outlined that ‘the stage (pre- or post-partum) at which the foetus/baby is attributed with human status’ is a central question that varies widely across different cultures and historical moments (Cecil 1996, 1). Anthropologists and other social scientists have consistently demonstrated variation in ascriptions of foetal and infant personhood. In some contexts miscarried foetuses are seen as ‘matter out of place’ rather than beings (Murphy and Philpin 2010, 535).

Bureaucracy is typically put in place to protect the system. In the aftermath of the Alder Hey organs scandal and the public environment of sensitivity towards the handling, retention, and disposal of remains and pregnancy tissue, bureaucracy surrounding pregnancy tissue disposal provides protection for the organization. The problem is not entirely the bureaucracy itself, but instead its inflexibility and prescription. Elsewhere I have suggested introducing a person-centred approach to pregnancy remains disposal that accommodates a diverse range of approaches and limit challenges to a woman’s experience of and agency about their body, their pregnancy, and their pregnancy material (Kilshaw 2024a). This would be a flexible system to allow for women learning as much or as little about disposal options and includes an opt out for those who wish to move on and/or do not wish to engage with navigating the ontology of their pregnancy material.

### Chronology and the miscarriage journey

Whilst miscarriage is typically represented as a swift and spontaneous event, it is common for a miscarriage to unfold over days if not weeks. The sequence of events and timing may impact how a woman understands her pregnancy material. Two main themes are chronology and ontology: stage in miscarriage trajectory, duration of pregnancy, and notions of foetal personhood. Surgical management of miscarriage typically follows unsuccessful expectant and/or medical management meaning women often encounter discussions around disposal weeks after their miscarriage diagnosis, which they commonly describe as the ‘moment of loss’:

It’s just something that you’ve maybe moved on from… they take you back… reminders of …that moment of loss. That’s what drags you back… for me it was so far down the line (Meredith, 35, first pregnancy, 8 weeks gestation).

It might have been five weeks from the point where I had a scan to when I had the surgery. By the time I had the surgery, I already felt like I had processed it. I think I felt like I was over it. Just wanted to move on and wanted it to be done with (Nell, 30, first pregnancy, 12-week pregnancy)

Meredith and Nell suggest that they experienced loss and upset at the time of diagnosis and in the subsequent days but had somewhat ‘moved on’ by the time of their surgery. For many women a miscarriage may be a drawn-out episode at the point of the surgical removal of the tissue, they are weary and ready to conclude the chapter.

In the period between the diagnosis of miscarriage and the surgical procedure women’s feelings of upset and loss may transform as may their understanding of the pregnancy material. In this context, women encountering discussions of disposal may have begun to frame the material as tissue. Furthermore, the material is framed as something needing to be removed to conclude the experience and to recover. Many women wish to become pregnant again and, thus, the removal of the tissue represents a necessary stage in their continuing quest for conception. The passage of time informs women’s framing of the miscarriage and its material. Ruth (47, third pregnancy, first miscarriage, 7-week pregnancy) explains,

By the time I went to [the hospital] it didn’t feel like a pregnancy anymore. It felt more like a medical procedure. It was about my body.

Meredith’s miscarriage was drawn-out, taking six months to complete. The first month following the diagnosis found her waiting to miscarry without medical intervention and the following months involved a series of interventions. I was first in contact with Meredith three months after her miscarriage diagnosis and during a period when she was dealing with ongoing health problems linked to it. Whilst she thought the miscarriage was complete, she learned that she had ‘retained tissue’ and had to undergo a surgical procedure to remove it. I met her again when she arrived at the clinic for a manual vacuum aspiration (MVA), a surgical management, and I observed as the clinician discussed her disposal options. Meredith later described how she felt in that moment,

Then to have the consent… to talk about the remains… it really threw me… because I think for a while now it hasn’t been a pregnancy for me, it’s been an illness, a chronic illness that I’ve been dealing with and trying to get better from.

For Meredith, like Ruth, the focus had shifted from the pregnancy, as something separate, to her body. The focus is on medical procedures sought to improve her health. The pregnancy tissue becomes something that needs to be removed to return to a state health for Meredith and other women who undergo surgical management. The material is understood as harmful and unwanted that needs to be eliminated. The removal of the pregnancy tissue is a necessary therapeutic process required for full recovery and the conclusion of the episode and, thus encounters with hospital practices diverge from how they frame the material.

### Classification of material: developing foetal personhood

Women understand their pregnancy material differently from that suggested by clinical practices. Upset and feelings of loss in early miscarriage are often disentangled from the tissue of that pregnancy whilst clinical procedures for disposing of pregnancy remains suggest their fusion. When Alex encountered the discussion about disposal prior to surgery, she had processed the experience:

In my head I was done with it and then I started having to think about it.

The procedure forced her to re-engage with the experience of loss, yet this is disengaged from the physical material:

There wasn’t a real emotional attachment, that’s not to say I wasn’t upset, there was a lot of crying and upset about what could have been but not really upset about [what it] was at that point.

Whilst sad about the end of her pregnancy, Alex did not frame it as a ‘baby loss’ but the loss of ‘what could have been’. Her focus was the concept of the pregnancy not the potentiality of the material itself: the material was not the fulcrum of loss.

Divergence in approaches to pregnancy tissue revolve around notions of value and are tied to understandings of developing personhood. Women suggest certain plot points, which impact their experience of pregnancy and their sense of loss when it ends (Middlemiss and Kilshaw 2023). A change in approach to pregnancy often follows the dating scan, which typically occurs around 12-week gestation. Seeing the (active) foetus and hearing a heartbeat is described or anticipated as a significant moment, after which women begin to understand the foetus as a baby. Nell resisted the framing of her miscarriage as the loss of a baby. During our second interview, Nell was pregnant and reflected on her experience considering her developing pregnancy:

The [miscarried pregnancy] was just a bunch of cells… Whereas now I’m pregnant and I can feel her moving and I know that she looks like a baby and at the 12-week scan she looked a baby. I was like, *Hmm I wonder how I’d feel about that consent form if I was shown that at 24 weeks?* I’m sure I would feel completely different about it… and I remember us talking about that there should be two different forms. I feel really strongly about that now… they need to be completely different because what’s inside you is completely different. So, I feel quite strongly about that… If its someone that’s going through it at seven weeks, eight weeks, and someone that’s going through that at 20 weeks, they’re two completely different experiences and should be dealt with separately.

Nell notes that ‘seeing her baby’ during her 12-week ultrasound scan and feeling her move impacts the way she thinks of her. Seeing the foetus and particularly something that resembles a baby is central to notions of developing foetal personhood. Rose (40, fourth pregnancy, third miscarriage, 12-week pregnancy) compares her experience to friends who had experienced pregnancy loss later in their pregnancy:

For me it was too early for anything… it wasn’t far enough ahead in my mind to be a baby… I know people that have lost babies at 20 weeks, for me that is more physically a baby then, it looks like a baby… whereas, at the stage I’ve been losing them it doesn’t really look like anything.

Many women experiencing first trimester miscarriage had not seen the (active) foetus or heard a heartbeat and, thus, understood their pregnancy as containing what might *become* a baby. As Scarlett (32, first pregnancy, 7 weeks pregnancy) explained,

So then being confronted with… that consent form and the treating of it as more than a small clump of cells… it made it a lot more upsetting because it makes it a baby, which isn’t how I had been thinking of it…. But there was one policy and one size fits all. And if you were 20 weeks, I am sure it would be a baby, you’ve heard a heartbeat, you’ve done scans, you probably know the gender, like, it is a baby. For me I had never heard a heartbeat, it was too early and part of the way I was coping is it’s not a baby. So being asked to treat it like it was a baby suddenly became very upsetting.

During our second interview, Scarlett expanded on the divergence between how she understood her pregnancy and that of the hospital bureaucracy:

I had how I was framing it and to be forced to frame it in a different way can make it more traumatising than it is. If you have to see it through that lens and then say, oh…and then treat it like… a bay. and it’s not… at 7 weeks it is a cluster, it is barely… definitely not even viable not even in the crazy US nonsense that is going on. It’s not a baby and I shouldn’t be forced to confront it like that. It is hard enough to confront it for what it was then to have to add all that personhood on top.

Scarlett raises the issue of personhood and dismisses the notion that her 7-week gestation pregnancy contained a baby. She refers to the 2022 change in abortion laws in the United States as being relevant to ascriptions of personhood. Scarlett was not the only woman to contextualise her pregnancy end in relation to diminishing reproductive rights in the US. The reversal of Roe v. Wade ended the federal right to an abortion in the United States (Buchbinder et al. 2022). Informed by her worldview, Scarlett’s approach to her pregnancy and its material is challenged by the clinical procedures, which force her to see it through a different lens that conflicted with her own understanding of it. Scarlett experiences this as destabilising and distressing,

One of the ways I had been coping with the miscarriage, it was very early, I was maybe six or seven weeks. For me… with my personal belief system… is it’s not a baby. It’s a clump of cells that has a lot of hopes and dreams attached to it and it could be a baby, but it’s not a baby… So then being confronted with … that consent form and the treating of it as more than a small clump of cells, was just, it made it a lot more upsetting because it makes it a baby, which isn’t how I had been thinking of it…. So being asked to treat it like it was a baby became very upsetting (Scarlett, 32, first pregnancy, 6–7 weeks gestation).

Nina (37, 3-year-old son, fifth pregnancy including an ectopic pregnancy, third miscarriage of a 7-week pregnancy) similarly explained that she didn’t understand her miscarriage to be the loss of a baby:

I felt that… it’s not that something wasn’t alive, but it certainly wasn’t a baby to me at that point… it wasn’t a child. We didn’t feel that there was a need for a more formal ritual around a goodbye… a burial or cremation. … So, yes, I suppose there was a surprise about the first options I suppose just because for me it wasn’t a baby.

Nell (30, first pregnancy, 12-week pregnancy) who had reflected above the developing personhood of her current pregnancy described her miscarriage in a similar way to Nina,

Treat it like any other kind of thing that you’re removing from your body… maybe the part of me trying to process it was me being like, this wasn’t a baby. It was never going to work. So, don’t think about it as a baby. It’s literally just some cells and tissues. So, then to be told, ‘Well, you know, we’re going to bury it’. Treat it like it is a human being is like totally opposite to how I’m trying to process it.

Nell begins by framing pregnancy remains as her tissue, the same as other material removed from a body; a viewpoint that is aligned with the legal classification of pregnancy tissue as being tissue of the woman. She then continues to make sense of this approach by suggesting perhaps this was part of the way she was navigating the experience. She was not thinking of the material as a baby, but rather ‘cells and tissues’ and suggests, as does Scarlett that this approach may be part of a protective mechanism to shield her from feelings of (baby) loss. These women then experience encountering hospital practice of burying the pregnancy remains as problematic, as it frames it as a corpse and makes assumptions about a bereavement response. In this way, hospital practices challenge women’s measures to guard themselves from potential grief.

Scarlett had decided to undergo medical management of her miscarriage, which would have meant her passing the pregnancy material at home. However, when this process was not successful, she required surgical management to eliminate the tissue and complete the miscarriage. She points out the contradiction in approaches to disposal,

I didn’t know I was going to be asked the question and I suppose given that they… had been very happy to send me home and to let me pass it into a toilet and flush it and that be it. To then be asked which of these funeral options essentially, I wasn’t prepared.

She highlights that when she had been administered medical management there was no discussion about pregnancy remains disposal and she was free to treat her pregnancy material as she wished. Yet when she required intervention in the clinic, her ability to treat her pregnancy tissue as waste was effectively removed: location dictates how the pregnancy materials are treated with women in the clinic having to encounter discussions and decisions about disposal.

### Classification of value: absence of incineration

Whilst women in the clinic are given choice as to how their pregnancy materials are disposed of, all options in the trust where I conducted my research were a form of ceremonial disposal, which involve cremation or burial typically accompanied by a ceremony with features of an abbreviated funeral (see Kuberska 2020)^6^. There was no option for women like Scarlett who prefer their pregnancy material to be treated as waste: such a disposal pathway was not an option in keeping with UK practices. Disposing of pregnancy remains *via* a clinical waste route became seen as unacceptable in the past decade (Kilshaw 2024b) although legal. Guidance suggests that ‘sensitive incineration’, which involves the separation of the material from clinical waste throughout the disposal pathway before being incinerated separately, is acceptable. Whilst the HTA and Royal College of Nursing regard sensitive incineration as an appropriate method and recommend it being offered to women as an option, the DBB research team found confusion with most trusts no longer offering incineration (McGuinness and Kuberska 2017). In cases where incineration was available it was offered only in specific circumstances, such as when there was no identifiable foetal tissue, for terminations, for early gestational stages, or in cases of absence of decision from the woman (McGuinness and Kuberska 2017). In Scotland incineration of foetal tissue is not banned but is considered inappropriate in any circumstance (HTA 2021).

Despite the explicit permission for sensitive incineration as a legitimate method, some trusts misinterpreted this point in the guidance; it may be that incineration is felt to be an inappropriate disposal method and documentation suggesting that incineration was incompatible with treating foetal remains with dignity was found (McGuinness and Kuberska 2017, 16). This resonates with interviews with health professionals, with some suggesting the disposal in a respectful and sensitive way was a legal (see below for further discussion) but also a moral and ethical issue. The controversies mentioned above have resulted in a general perception of incineration as a socially unacceptable method of disposing of foetal remains (McGuinness and Kuberska 2017, 17); in particular, memories of the Dispatches documentary, which referred to incineration as ‘burning babies’ have likely resulted in a risk adverse approach to avoid attracting the ire of the vocal cohort of advocates who equate pregnancy loss with the death of a baby. Many DBB interviewees mentioned the scandals surrounding the incineration of early pregnancy losses despite parents being informed they would be cremated (McGuinness and Kuberska 2017, 17). The emphasis was placed on incineration as inappropriate rather than the misinformation about the mode of disposal, which ‘highlights a generally unfavourable attitude to this option of disposal’ (McGuinness and Kuberska 2017, 17). Funerary industry persons suggest sensitive incineration was not considered an appropriate disposal method for pregnancy remains, unlike either cremation or burial (McGuinness and Kuberska 2017, 17), a finding supported by my research. The role of the chaplaincy in revisions of pregnancy remains disposal practices at local NHS trusts may also have informed decisions about the inappropriateness of incineration as a mode of disposal.

That some trusts do not offer incineration ‘evidences a clear gap between about the day-to-day reality of trust practices and the expectations contained in the HTA (2015), RCN (2015), and Sands Guidance (SANDS. 2016) that women be provided with information on all disposal options’ (McGuinness and Kuberska 2017, 16). Caroline Browne, the previous Head of Regulation at the HTA, notes the omission of sensitive incineration as a mode of disposal ‘warrants careful consideration, particularly as the lack of availability of incineration may conflict with a woman’s view about the status that her pregnancy remains should be accorded’ (Browne, quoted in McGuinness and Kuberska 2017). Incineration may be an important option for women including some of those experiencing an elected termination (Myers, Lohr, and Pfeffer 2015) or the loss of a wanted pregnancy who would not find ceremonial disposal acceptable (McGuinness and Kuberska 2017), as my research supports (Kilshaw 2024a, 2024b) and as evidenced by Scarlett:

Maybe it needs to be up to a certain point in gestation … the right to have it treated as medical waste. Because I’m very uncomfortable with the way that it was handled.

At the heart of this is that disposal practices construct pregnancy material as a baby, which contradicts how many women understand them

## Clinical practices and defining normativity

Clinical practices have implications for normativity: participants often interpreted the practices to be reflective of most women’s needs. Caregiving in clinical settings align pregnancy loss as bereavement (Fuller and Kuberska 2022, Kilshaw 2024b) leading some women who do not frame their miscarriage in this way to feel deviant. Leslie Reagan (2003, 369) notes that contemporary clinical approaches in the US treat women’s responses and emotion in a formulaic way and participate in establishing norms for female behaviour and emotions. Furthermore, these norms are rooted in conservative gender ideology that ‘treats motherhood as women’s greatest achievement and its denial as women’s greatest suffering’. Clinical practices dictate how women should respond to pregnancy loss (Reagan 2003, 369) and are insensitive to some women and their response to miscarriage. Such practices are in keeping with an intensive parenting culture, which stresses personal fulfilment through parenthood. In such contexts the social status of being a parent has been amplified (Faircloth and Gurtin 2017), which encourages the maintenance a maternal identity in the face of pregnancy loss. Such an approach is supported by pregnancy loss advocacy groups which has informed clinical approaches. Given hospital procedures are likely to provide legitimation of approaches as normative, it is unsurprising that some women surmised that such practices reflected the norm or were created to serve the majority. The three options presented to women during the discussion and consenting procedure all involve ceremonial disposal. Indeed, the only way a woman could opt out of ceremonial disposal would be to take the material home and treat it as waste.

An important element in normativity is the way discussions around disposal and consenting were entangled with implications of legality. Consent for pregnancy remains disposal takes on the connotation of other kinds of surgical procedures and legalistic associations of a contract. Indeed, in most cases the consent for remains disposal occurs at the same time or soon after the consenting process for the surgical procedure leading to understandable leakage of meaning. It was common for patients and staff alike to refer to the consent form as a legal document and the process as being required for legal reasons. Indeed, references to legality also provide a means to excuse or shift attention away from the procedure, suggesting it is not governance created by the hospital but a governing establishment above.

Alex assumed the consenting process was tied to legality, asking me if it was ‘a legal thing’, and leading her to question her response and her understanding of the pregnancy material as medical waste. The experience made her feel,

Like an unemotional weirdo… that all made me feel a bit like a monster. … I just thought that is what woman want… to the point of not being normal.

Alex interpreted hospital practices as being responsive to what most women wanted: framing the experience as the loss of a baby requiring some form of funeral. This led Alex to understand her response as peculiar. Nell similarly felt the practice might lead women to surmise that they aren’t ‘processing this the right way’ and that something was wrong with their approach, leading them to ask, ‘Why am I not thinking about this like it was a baby?’ Whilst forcing women to negotiate and question the way they understand the ontology of the pregnancy material is problematic, what is particularly so is that most women do not actively choose ceremonial disposal.

There was no option for women in the clinical setting to choose non-ceremonial disposal, such as sensitive incineration or a waste pathway, as Scarlett pointed out. A small number of women passed their pregnancy in the hospital bathroom: Pregnancy material that emerged and passed into the toilet or placed into a bin was routinely retrieved by staff. Several staff reported an incident when a doctor, new to the Trust, had placed pregnancy tissue in the clinical waste bin much to the horror of the other staff members. A nurse retrieved the material. The incident caused friction but was explained as a cultural misunderstanding due the clinician being new to the country and to the trust.

The question of what is normative implies measurement against others. In seeking to understand the normative women compare themselves and their approach with those of other women, as described above. Alex (37, first pregnancy, 7 weeks gestation) explains that up until the point of being handed the information sheet about disposal options, she ‘was fine with it’ but the experience called into question the value of the material and the gravity of her sorrow. As Alex explains,

I’m the odd one out here then it makes sense. Like I almost- feel a bit like, not like a psychopath but like you know what I mean? Like an unemotional weirdo that I don’t have that emotional attachment to it. So, maybe I’m weird and that’s completely normal.

Clinical practices lend legitimacy to ways of constructing pregnancy material and frame these as the moral, right way to approach these materials. It is not surprising, then, that women, like Alex, who do not frame pregnancy tissue in this way will conclude that their response is abnormal. During our second interview, Alex continued to reflect on her response,

I knew instinctively like none, I don’t know just knock me out, deal with it and then leave me go home. And that just made me feel maybe that’s not the right reaction because of those options…. it made me feel again a bit like maybe this is not quite normal how I react.

Alex had assumed that her pregnancy tissue would be treated as medical waste, which resonated with her understanding of the material. When she discovered that this was not the case and that there was only a means to bury or make private arrangements, she interpreted her understanding the material as an incorrect and her response to the experience as abnormal. Just as Alex compares herself to others in seeking the normative, other women sought to understand what women typically do when in their situation. A standard response when confronted with the form was ‘what do most women do?’. In the often unexpected and unfamiliar experience of miscarriage, women sought to know what other women did. Clinical practices structure normativity. When presented with the option women typically assumed the clinical practices reflected what most women wanted: ceremonial disposal and/or a form of memorialisation.

## Conclusions

This paper has contributed to the fundamental question in medical anthropology about what is a person and who has the power to define this. The framing of miscarriage and pregnancy tissue shift and change depending on the cultural and historical context and women’s responses are diverse. Current clinical practices toward the disposal of pregnancy remains were developed in response to a cultural shift in how society views pregnancy loss with such practices part of a dedication to approaching miscarriage with increased sensitivity. The paper has shown how caregiving in clinical settings in England is structured by an understanding of miscarriage as bereavement and an element of this is approaching pregnancy material as though it were human remains or a corpse. In so doing, clinical practices produce foetal personhood, which may not be in keeping with women’s notions of their pregnancy and pregnancy material. It is integral to be vigilant about such processes, as clinical practices and the meaning they contain become legitimated and lend authority to ways of constructing pregnancy material and frame these as the moral, right approach. In this case, consent for ‘pregnancy remains disposal’ has connotations of other surgical procedures and legality, which lends further weight to notions of normativity.

The way pregnancy remains are handled is informed by notions of value, import, and worth. Location informs the choices, requirements to act, and governance confronting women experiencing a pregnancy end. Governance may become focused on protecting the institution from scrutiny or scandal rather than on care. The paper has shown how women experience one element of sensitive care, discussions around pregnancy remains disposal, as insensitive. The research suggests that framing the process as consent constrains and compels the exchange in a way that may cause discomfort and distress (Kilshaw 2024a). Hospital practices compel women to encounter their pregnancy tissue and to make moral decisions about its ontological status in a way that is not always present in domestic settings. Approaches to pregnancy material will continue to transform as a result of lived experience and the shifting political and cultural landscape. With this fluid terrain, clinical practices require increased flexibility to provide for diversity and nuance in women’s approaches to their pregnancy end and material. As Scarlett said, ‘There should be a breadth of options. Like all things to do with reproduction there should be choice.’

## Notes

1 The NHS is divided into regional areas. An NHS trust is an organisational unity within the NHS services of England and Wales that serve either a geographical area or specialised function. In this case it involved several hospitals and clinics.

2 The research was suspended between June 2021-May 2022 due to the COVID-19 pandemic.

3 This number represent the women I have interviewed in the UK over the past 10 years for the current and previous research project.

4 During COVID-19 restrictions, surgical options were limited, which likely impacted my observation that most women who were undergoing surgical management had tried other management options prior to their surgery.

5 The NHS is divided into regional areas. An NHS trust is an organisational unity within the NHS services of England and Wales that serve either a geographical area or specialised function.

6 Most pregnancy tissue burials were not accompanied by a funeral service, but could be seen as ceremonial in that they were transported and overseen by a funeral director and buried in the same Sands memorial garden as the witnessed and chaplain-led burials, which happened soon after. In many NHS trusts cremation or burial of pregnancy remains typically includes elements of a funeral, such as a blessing or reading(s).
